# Rural-Urban Disparity in Premature Cancer Mortality in Young People Aged 15–44 Years in China, 2004–2021

**DOI:** 10.3389/ijph.2025.1608133

**Published:** 2025-02-12

**Authors:** Chunrong Chen, Xing Xing, Shaojie Li, Bo Qu, Chunyu Liu, He Zhu

**Affiliations:** ^1^ Department of Radiation Oncology, The First Affiliated Hospital of Qiqihar Medical College, Qiqihar, Heilongjiang, China; ^2^ School of Public Health, Peking University, Beijing, China; ^3^ China Center for Health Development Studies, Beijing, China

**Keywords:** rural-urban disparity, cancer, mortality, young people, age-standardized mortality rate

## Abstract

**Objective:**

This study aims to examine and compare premature cancer mortality in young people aged 15–44 years old between rural and urban areas to inform early-onset cancer prevention.

**Methods:**

The data were obtained from the China Death Surveillance Datasets from 2004 to 2021. The study sample consisted of cancer deaths of young people aged 15–44 years old. Age-standardized mortality rates (ASMRs) were calculated, and joinpoint regressions were used to examine trends in ASMRs.

**Results:**

There were overall decreasing trends in ASMRs for all cancers in both rural and urban young people in China from 2004 to 2021. However, the decrease was relatively slower in rural areas, where ASMRs for pancreatic and ovarian cancers showed increasing trends. The five leading types of cancer deaths consistently remained liver, lung, leukemia, stomach, and other cancers in both rural and urban areas after 2013.

**Conclusion:**

Our findings indicate that there were rural-urban disparities in cancer mortality in young people, which showed a different pattern compared to other age groups. More efforts are needed to develop effective early-onset cancer prevention strategies, with particular emphasis on liver cancer and rural areas.

## Introduction

Concerns regarding early-onset cancers in young people are on the rise both in China and globally. According to estimates from the Global Burden of Disease (GBD), the global incidence number of early-onset cancers reached 3.26 million in 2019 with a 79% increase from 1990, and the number of early-onset cancer deaths rose by approximately 28% between 1990 and 2019 [1]. Early-onset cancers could potentially lead to premature death, poor quality of life, family financial burden, and healthcare system burden [2, 3]. The patterns of cancer in young people may differ from those in other age groups and may vary between rural and urban areas [4]. For example, rural communities experience higher death rates from lung, cervical, and colorectal cancers than urban communities because of poverty, health risk behavior, and lower vaccination and screening rates [5, 6]. In addition, leukemia and lymphoma are relatively common in adolescents, while lung and colorectal cancers are more prevalent in older populations [7, 8]. However, few studies have focused on cancer patterns and rural-urban disparity in young people, particularly in China. Furthermore, young people often fail to recognize the potential seriousness of early symptoms, which can result in delays seeking medical treatment until the cancer has progressed to advanced stages, thereby exacerbating the overall disease burden [9]. Therefore, there is a clear need to enhance our understanding of the epidemiology of early-onset cancer and mortality in young people.

In this study, we examined and compared the trends and patterns of premature cancer mortality in young people aged 15–44 years old between rural and urban areas in China.

## Methods

### Data Sources and Study Sample

The data were obtained from the 2004–2021 China Death Surveillance Datasets (CDSDs) [10, 11]. The CDSDs are publicly available and are a primary nationally representative source of the mortality data in China. The CDSDs included 161 monitoring sites between 2004 and 2012, covering 31 provinces (autonomous regions and municipalities) and accounting for 6% of the national population. Since 2013, the CDSD has started the data expansion to include 605 monitoring sites, accounting for about 24% of the national population. In order to address underreporting problems and improve the data quality, the data excluded monitoring sites with a ≤3% mortality rate between 2004 and 2012, a ≤5% mortality rate in 2013, and a ≤4.5% after 2013 [10, 12]. The data also excluded several sites, which could potentially affect the overall results.

In this study, we focused on the sample of cancer deaths for young people aged 15–44 years old. We identified 17 subtypes of cancer based on the International Classification of Diseases, Tenth Revision, Clinical Modification (ICD-10-CM): lips/mouth/pharynx (C00-C14), esophagus (C15), stomach (C16), colorectal (C18-C21), liver (C22), pancreatic (C25), lung (C33-C34), skin (C43-C44), breast (C50), cervical (C53), uterine (C54-C55), ovarian (C56), prostate (C61), bladder (C67), lymphoma (C81-C90, C96), leukemia (C91-C95), and other cancers (cancers not specified above in C00-C97, and detailed codes in Supplementary Table S1) [10].

### Measures

Age-standardized mortality rates (ASMRs) for overall and specific types per 100,000 people in the total young population, stratified by urban and rural areas, were calculated from 2004 to 2021. The ASMRs were adjusted based on the World Segi’s population structure using the direct calculation method. The rural/urban mortality ratios (R/U ratios) were calculated by dividing the rural cancer ASMRs by the urban cancer ASMRs.

### Data Analysis

Descriptive analyses were first used to characterize the distribution of specific cancer types among all cancer deaths stratified by rural and urban areas for 2004, 2012, 2013, and 2021. We selected the years of 2012 and 2013 at midpoints because the dataset expanded its sampling areas in 2013. Next, we calculated ASMRs stratified by cancer type, year, and rural and urban areas, and we also described the trends in ASMRs.

We conducted joinpoint regressions to estimate the average annual percentage change (AAPC) in ASMRs, which was calculated by averaging the annual percent changes (APCs) derived from the joinpoint regression analysis, with each APC weighted according to the duration of its respective segment within the time interval. Joinpoint regression has been widely used and well-suited for analyzing trends in time series data, particularly in the context of public health data, such as cancer mortality rates [13, 14]. We also used the change-point analyses to identify thresholds between ASMRs and years to improve the stability of the results and address the sampling expansion issue. All statistical analyses were performed using R software (version 4.3.0; R Foundation for Statistical Computing, Vienna, Austria) and Joinpoint software (version 5.0.2, Applications Branch, National Cancer Institute, United States). A two-tailed p-value <0.05 was considered statistically significant.

## Results

### Distribution and Ranking of Cancer Deaths in Young People in Rural and Urban Areas

**FIGURE 1:**
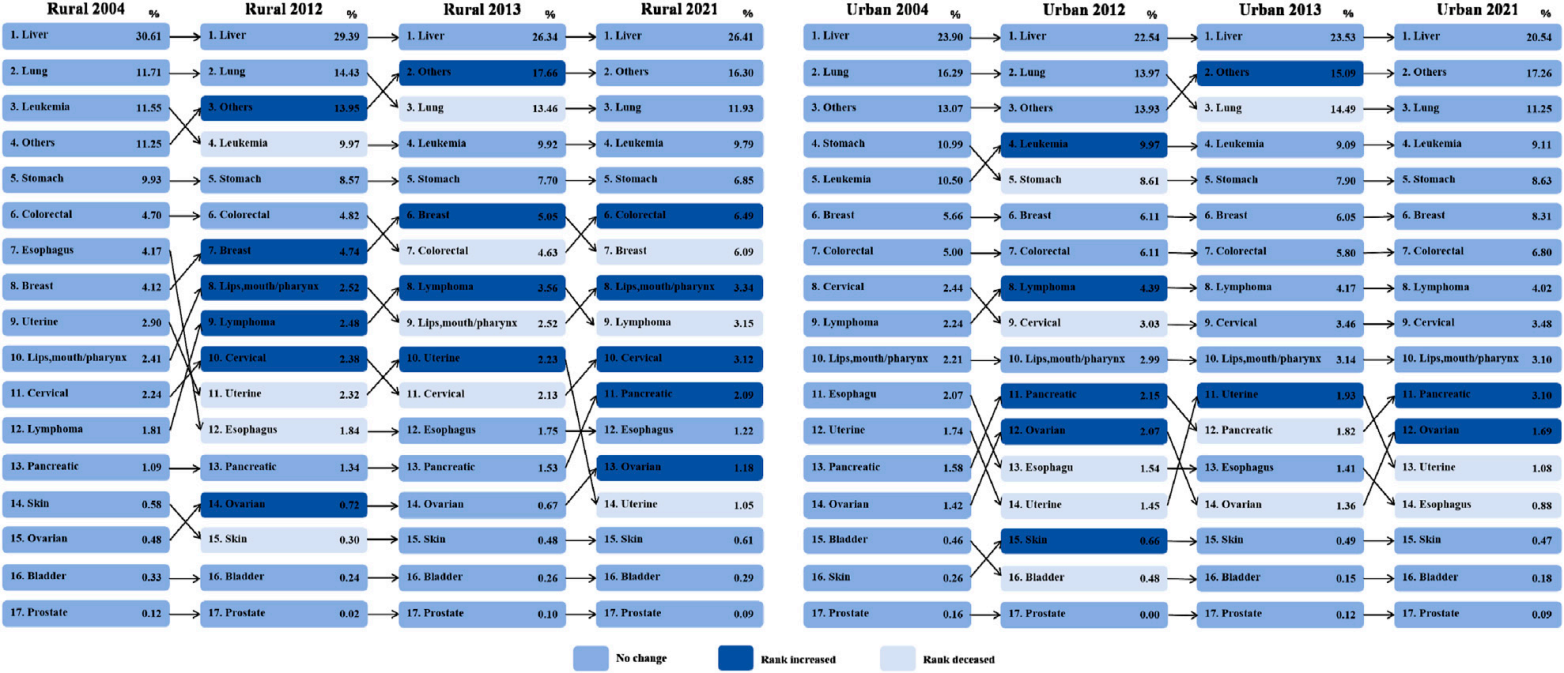
Distribution and ranking of cancer deaths in young people aged 15–44 years old stratifed by rural and urban areas, China 2004–2021. Note: Preliminary ranking results for several low-ranking cancers should be interpreted with caution due to the small sample size.

Of the sample aged 15–44 years old, the distribution of cancer death types remained relatively stable, particularly in urban areas (Figure 1). The five leading cancer types among cancer deaths were liver cancers, lung cancers, other cancers, leukemia, and stomach in both rural and urban areas across years, collectively representing approximately 75% of cases. For example, in 2021, liver cancers (Rank 1st) accounted for 26.41% of cancer deaths in rural areas compared to 20.54% in urban areas; leukemia (Rank 4th) accounted for 9.79% in rural areas and 9.11% in urban areas. Additionally, cancer deaths exhibited a different ranking pattern following the five leading types between rural and urban areas.

### Rural-Urban Comparison of ASMRs for Cancers in Young People

In rural areas, the ASMR for all cancers in young people decreased from 25.03 in 2004 to 20.10 in 2012 before the dataset expansion and decreased from 19.06 in 2013 to 14.97 in 2021 after the expansion (Table 1). In urban areas, the ASMR for all cancers in young people decreased from 22.44 in 2004 to 13.08 in 2012 before the dataset expansion and decreased from 15.84 in 2013 to 10.77 in 2021 after the expansion.

**TABLE 1:** Rural-urban comparison of age-standardized mortality rates (ASMRs) for cancers in young people aged 15–44 years old, China 2014–2021.

Cancer	ASMR 2004		ASMR 2012		ASMR 2013		ASMR 2021		R/U ratio 2004	R/U ratio 2012	R/U ratio 2013	R/U ratio 2021
	Rural	Urban	Rural	Urban	Rural	Urban	Rural	Urban				
Total	25.03	22.44	20.10	13.08	19.06	15.84	14.97	10.77	1.12	1.54	1.20	1.39
Liver	7.63	5.26	5.69	2.89	4.84	3.59	3.80	2.09	1.45	1.97	1.35	1.82
Others	2.86	3.30	3.00	1.88	3.50	2.49	2.59	1.96	0.94	1.59	1.40	1.32
Lung	2.92	3.59	2.74	1.76	2.41	2.19	1.72	1.16	0.81	1.56	1.10	1.48
Leukemia	2.97	2.56	2.28	1.45	2.21	1.67	1.64	1.15	1.16	1.57	1.32	1.43
Stomach	2.48	2.42	1.68	1.10	1.41	1.23	1.00	0.91	1.02	1.52	1.14	1.10
Colorectal	1.16	1.12	0.98	0.79	0.88	0.91	0.96	0.72	1.04	1.25	0.97	1.33
Breast	1.03	1.24	0.91	0.76	0.91	0.92	0.87	0.84	0.83	1.19	0.99	1.03
Lymphoma	0.46	0.52	0.53	0.61	0.73	0.71	0.50	0.47	0.87	0.87	1.03	1.07
Lips/mouth/pharynx	0.60	0.49	0.50	0.38	0.48	0.48	0.49	0.32	1.22	1.33	1.01	1.50
Cervical	0.56	0.53	0.46	0.39	0.39	0.53	0.45	0.35	1.04	1.17	0.74	1.27
Pancreas	0.26	0.34	0.27	0.28	0.28	0.28	0.30	0.32	0.77	0.96	1.00	0.96
Ovarian	0.12	0.32	0.14	0.26	0.12	0.21	0.18	0.19	0.38	0.53	0.58	0.95
Esophagus	1.06	0.46	0.35	0.19	0.30	0.21	0.17	0.09	2.29	1.81	1.43	1.83
Uterine	0.72	0.39	0.46	0.18	0.41	0.29	0.15	0.11	1.88	2.49	1.40	1.34
Skin	0.14	0.06	0.06	0.08	0.10	0.08	0.09	0.05	2.50	0.78	1.30	1.70
Bladder	0.08	0.10	0.05	0.06	0.05	0.02	0.04	0.02	0.83	0.83	2.11	2.45
Prostate	0.03	0.04	0.00^a^	0.00^a^	0.02	0.02	0.01	0.01	0.75	0.00^a^	1.18	1.47

^a^ Being 0.00 due to keeping two decimals.

Although both rural and urban ASMRs generally showed decreasing trends, the R/U ratio increased, measuring 1.12 in 2004, 1.54 in 2012, 1.20 in 2013, and 1.39 in 2021. In 2021, the R/U ratios for esophagus cancer (1.83), liver cancer (1.82), skin cancer (1.70), and lips/mouth/pharynx cancer (1.50) were relatively high. In contrast, the R/U ratios for pancreas (0.96) and ovarian cancers (0.95) were both below 1. Additionally, the R/U ratios for lymphoma and bladder cancers increased to be more than 1 following the dataset expansion.

### Trends in ASMRs for Cancers in Young People Stratified by Rural and Urban Areas

**FIGURE 2:**
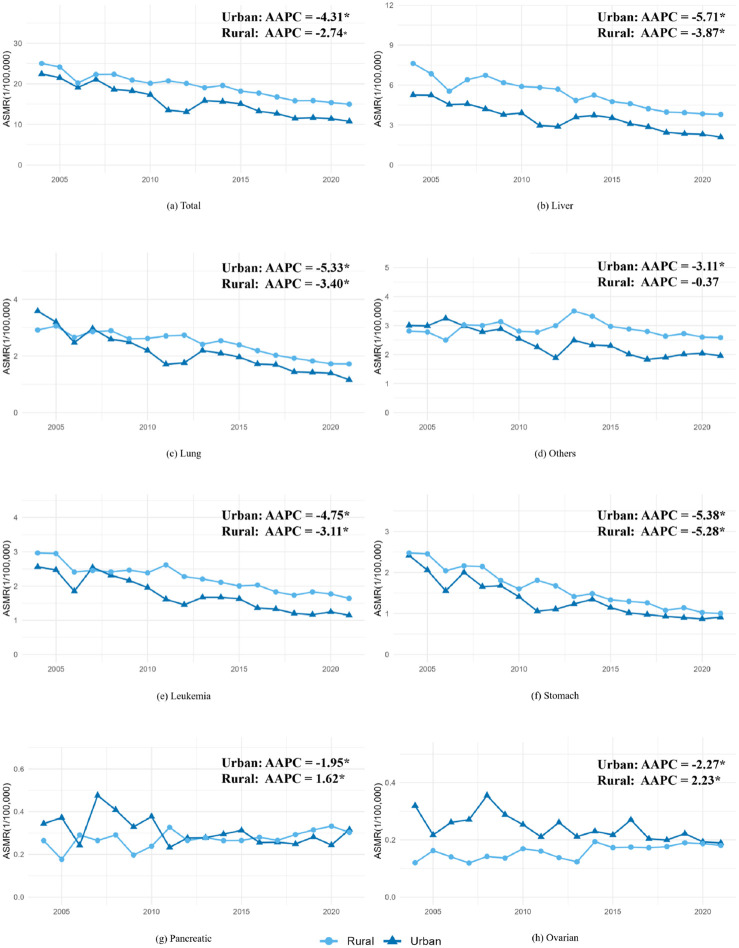
Trends in age-standardized mortality rates (ASMRs) for cancers in young people aged 15–44 years old stratified by rural and urban areas, China 2004–2021. *The average annual percentage change (AAPC) is significantly different from zero (*P* < 0.05).

Overall, the ASMR for all cancers in young people decreased by 46.41% (AAPC = −2.74, 95% CI: −3.21, 2.26) in rural areas and 70.21% (AAPC = −4.13, 95% CI: −4.90, 3.34) in urban areas between 2004 and 2021 (Figure 2). The ASMRs for liver, lung, leukemia, and stomach cancers decreased by approximately 50%–90% in both areas; however, the ASMRs for other cancers did not change significantly during this period in rural areas. Although the ASMRs for most cancers decreased, the ASMRs for pancreatic and ovarian cancers in rural areas increased by 27.54% (AAPC = 1.62, 95% CI: 0.17, 3.14) and 37.91% (AAPC = 2.23, 95% CI: 1.13, 3.34), respectively, from 2004–2021.

In addition, both joinpoint and changepoint regressions detected no significant changes in ASMRs for all cancers; however, they identified changes in ASMR for liver cancer in urban areas in 2010 and 2013, for lung cancers in rural areas in 2014, and for other cancers in rural areas in 2013 (Supplementary Tables S2, S3).

## Discussion

This study is among the first to examine premature cancer mortality in young people in China. The results indicate that liver, lung, leukemia, stomach, and other cancers consistently contributed to premature cancer mortality in both rural and urban young people in China from 2004 to 2021. Although the ASMRs for all cancers in young people showed decreasing trends, ASMRs for most cancers were higher in rural areas compared to urban areas, particularly for liver cancers. Furthermore, ASMRs for pancreatic and ovarian cancers in rural areas exhibited upward trends. These findings have important implications for informing the development of early-onset cancer prevention strategies.

Our findings indicated that there were decreasing trends in ASMRs for cancers in both rural and urban young people, but the decrease was relatively slower in rural areas. Similarly, a global study found that the morbidity rates of early-onset cancer increased from 1990 to 2019, while mortality rates and disability-adjusted life years (DALYs) slightly decreased [1]. Recent studies in Europe also exhibited potential differences in the prevalence of early-onset cancers between metropolitan and rural areas, with the geographical distribution identified as a risk factor [4, 15, 16]. This decrease in China may be attributed to government prevention efforts and advancements in cancer treatment. Both central and local governments have intensified their initiatives to implement cancer prevention and control actions of the Healthy China 2030 including expanding screening, improving early diagnosis and treatment, and developing standardized treatments [17]. Research indicates rural areas generally have lower health literacy compared to urban areas, which may contribute to higher mortality rates, primarily due to factors such as lower educational levels, socioeconomic disadvantages, and physical labor engagement. For example, a study reported that urban residents had higher rates of health-promoting behaviors and lifestyle habits (urban vs. rural: 14.03% vs. 7.33%), as well as higher rates of health knowledge and concept awareness (urban vs. rural: 34.50% vs. 20.42%) than rural counterparts in China [18]. The disparity between urban and rural areas may also be linked to elevated cancer incidence rates and prolonged periods before cancer diagnosis in rural areas [19]. With the anticipated rise in early-onset cancers, we recommend the implementation of more effective health interventions to improve public awareness regarding cancer prevention and routine screening, particularly among rural young people.

Our findings also identified potential trend changes in ASMRs, which disproportionally affect liver, lung, and other cancers in urban and rural areas in 2010, 2013, and 2014. The potential changepoint identified in 2010 for liver cancer deaths in urban areas may be related to the implementation of China’s basic public health policy in 2009 and free HBV vaccine programs [20, 21]. In 1992, the former Ministry of Health included HBV vaccines in the children’s immunization program, and the national HBV vaccination rate reached 70.7% in 1999 [21]. These changes could have contributed to early improvements in health outcomes, influencing the cancer mortality trends. The changes in 2013 and 2014 for urban liver cancer deaths and rural lung and other cancer deaths may be influenced by the CDSD expansion in China [11]. As mentioned in the methods, the CDSD monitoring sites reached to be 605 in 2013 compared to 161 in 2012 or earlier. Either 161 or 605 design is a nationally represent samples in China; however, due to limited experiences in collecting and managing the data, the new data may be affected. A study from China CDC as the data management agency examined the trends in chronic diseases in older people from 2004 to 2018, and it discussed that the data expansion in 2013 may suffer the underreporting issues to underestimate the mortality rate [12]. More studies are needed to monitor the trends and changes in early-onset cancer incidence and mortality rates.

Our findings revealed that the top five cancers leading to deaths in young people in China were liver, lung, stomach, leukemia, and other cancers, with stable ranking in both urban and rural areas, which showed a different pattern compared to other age groups. According to an estimate based on 700 registries reporting high-quality data on cancer incidence and mortality across China, the top five cancers leading to deaths were lung, liver, colon-rectum, esophagus, and other cancers in 2022 [22]. Notably, liver cancer and leukemia in young people were more likely to result in cancer deaths compared to in the general population or older population, especially in rural areas [23]. Infection with the hepatitis B virus (HBV), excessive alcohol consumption, and exposure to aflatoxin are recognized as significant risk factors for liver cancer [24]. HBV infection remains the predominant risk factor for liver cancer in China, accounting for 63%–67% of all liver cancer cases [25]. Although the prevalence of HBV has decreased due to the national immunization program, the HBV infection rate is higher in rural areas than in urban areas, which may contribute to increased cancer mortality for young people in rural areas [26]. Rural areas continue to carry the heavy burden of chronic HBV infection, and mortality from HBV-related liver cancer is expected to remain at high levels in the coming decades [26]. Therefore, more efforts are needed to enhance early prevention and detection for rural young people with HBV.

In addition, leukemia is one of the leading causes of cancer deaths among children and adolescents in China, and rural people generally experience higher mortality rates and disease burdens compared urban people, which aligns with our findings [27, 28]. Numerous studies have demonstrated associations between leukemia incidence and environmental exposure. Particularly among rural males employed in factories, significant exposures to ionizing radiation and benzene solvents during industrial production are closely linked with increased leukemia mortality rates [29, 30]. Prior studies have observed excess leukemia risks among individuals, engaged in agriculture or livestock farming who were exposed to pesticides and infectious pathogens [31]. Therefore, health education and necessary occupation protection should be implemented to reduce exposure to carcinogenic substances among high-risk populations at an early stage.

Our findings also indicate that pancreatic and ovarian cancer deaths have increased from 2004 to 2021 in rural young adults. Both pancreatic cancer and ovarian cancers are highly lethal malignancy, with delayed diagnosis being the primary reason for their high mortality rates. According to a clinical study conducted in China, fewer than 20% of pancreatic cancer patients are diagnosed in the early stages of their disease [32]. Early-stage pancreatic and ovarian cancers often present few obvious symptoms and typically become apparent only when the cancer has invaded surrounding tissues or organs at advanced stages [33, 34]. Approximately 70% of ovarian cancer patients were diagnosed at advanced stages, and there is currently a lack of effective early diagnostic techniques [35]. Alcohol abuse, smoking, and a high intake of processed meat have been shown to be associated with the development of pancreatic cancer [33]. Insufficient dietary fiber and vitamin intake, diabetes, and obesity are potential risk factors for ovarian cancers [34]. Promoting access to the routine screening and healthy lifestyles are essential for reducing the cancer risk and improving health outcomes, particularly for rural young people.

These findings should be interpreted with caution. First, the sampling expansion of the CDSD in 2013 may affect the continuity and comparability of data across years. Although the data collection agency implemented quality control procedures of excluding monitoring sites with low-quality data, there remains a possibility of the underreporting and underestimating cancer deaths. Several studies have examined the trends in deaths including periods before and after 2013; however, few of these studies, as well as no official reports, discussed the potential influences of the data expansion on the results. Second, the cancer death data in this study are cumulative data due to the availability of data from the CDSDs, and there is no individual-level information on socioeconomic, environmental, or behavioral factors that may contribute to or explain disparities. Future studies are needed to investigate the specific differences in cancer deaths between rural and urban young people. Third, despite multiple verification procedures to improve data accuracy, the diagnosing bias in cancer deaths may exist and vary across hospitals. Finally, the data did not allow for the specification of cases classified into other cancers, which may limit the interpretability of the results.

## Data Availability

The original data for this study can be accessed at: https://ncncd.chinacdc.cn/jcysj/siyinjcx/syfxbg/. The data used in this study was accessed on 10 March 2024.
