# Supplementary material for: Rural-Urban Disparity in Premature Cancer Mortality in Young People Aged 15–44 Years in China, 2004–2021
Source: Int J Public Health. 2025 Feb 12;70:1608133. doi: 10.3389/ijph.2025.1608133 (PMC11859585; doi:10.3389/ijph.2025.1608133)
Supplement: Supplementary file 1 [file DataSheet1.doc]

**Supplementary Materials**

**
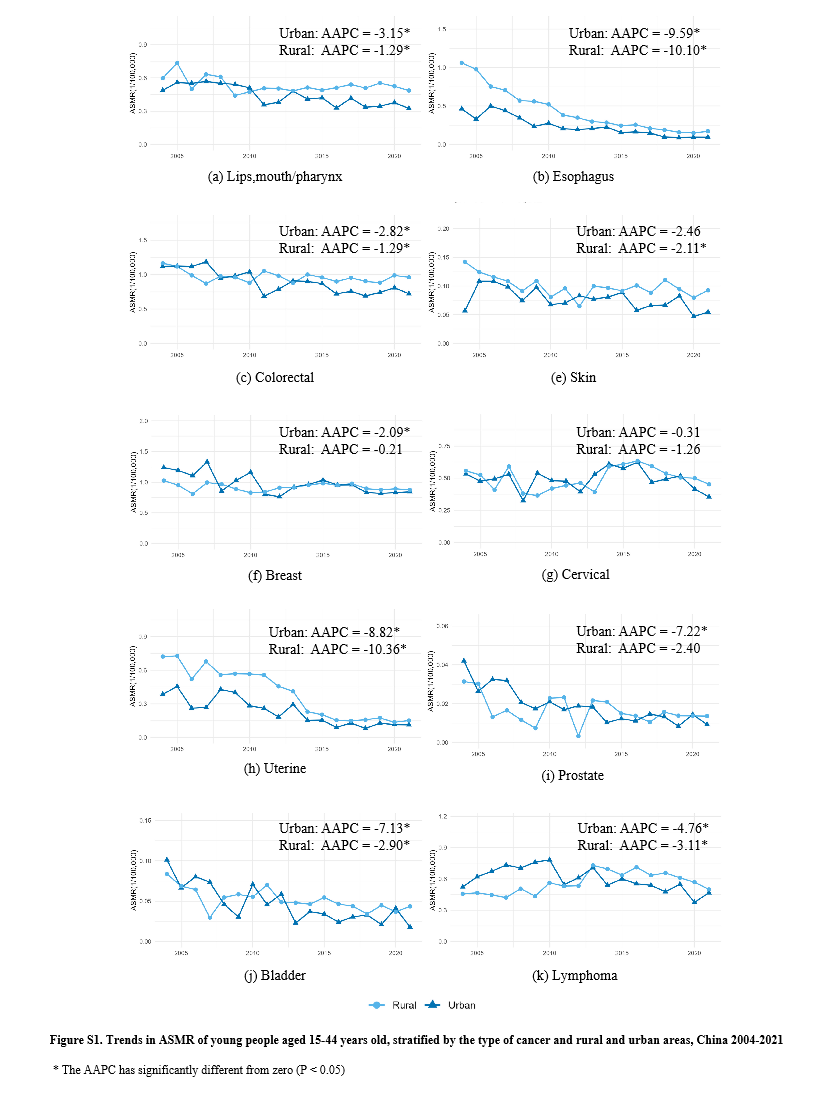
**

**Table S1. ICD-10-CM codes for classifying other cancers**

| **ICD-10-CM codes** | **Cancer site** |
| --- | --- |
| C17 | small intestine |
| C23 | gallbladder |
| C24 | other and unspecified parts of biliary tract |
| C26 | other and unspecified parts of digestive organs |
| C30 | nasal cavity and middle ear |
| C31 | paranasal sinuses |
| C32 | larynx |
| C37 | thymus gland |
| C38 | heart, mediastinum, and pleura |
| C39 | other and unspecified malignant neoplasm of respiratory system and intrathoracic organs |
| C40-C41 | bone and articular cartilage |
| C45-C49 | mesothelial and soft tissues |
| C51 | vulva |
| C52 | vagina |
| C57 | other and unspecified female genital organs |
| C58 | placenta |
| C60 | penis |
| C62 | testis |
| C63 | other and unspecified male genital organs |
| C64 | kidney (except renal pelvis) |
| C65 | renal pelvis |
| C66 | ureter |
| C68 | urinary tract |
| C69-C72 | eye, brain, and other parts of central nervous system |
| C73-C75 | thyroid gland and other endocrine glands |
| C76-C80 | unspecified, secondary, and unspecified site |
| C97 | multiple independent (primary) sites |

**Table S2. Joinpoint analyses of ASMR for cancers among young people aged 15-44 years old stratified by rural and urban areas in China from 2014 to 2021**

| **Type** | **Region** | **AAPC (%)** | **Trend1 years** | **APC (%)** | **Trend2 years** | **APC(%)** | **Trend3 years** | **APC (%)** |
| --- | --- | --- | --- | --- | --- | --- | --- | --- |
| Overall | Urban | -4.13 (-4.90, -3.34) | 2004-2021 | -4.13 (-4.90, -3.34) |  |  |  |  |
|  | Rural | -2.74 (-3.21, -2.26) | 2004-2021 | -2.74 (-3.21, -2.26) |  |  |  |  |
| Lips,mouth/pharynx | Urban | -3.15 (-4.37, -1.86) | 2004-2021 | -3.15 (-4.37, -1.86) |  |  |  |  |
|  | Rural | -1.29 (-2.40, -0.05) | 2004-2010 | -4.76 (-14.83, -1.36) | 2010-2021 | 0.65 (-0.90, 7.68) |  |  |
| Esophagus | Urban | -9.59 (-11.42, -7.65) | 2004-2021 | -9.59 (-11.42, -7.65) |  |  |  |  |
|  | Rural | -10.10 (-11.85, -9.19) | 2004-2019 | -11.70 (-17.51, -3.95) | 2019-2021 | 2.83 (-11.82, 11.86) |  |  |
| Stomach | Urban | -5.38 (-6.48, -4.24) | 2004-2021 | -5.38 (-6.48, -4.24) |  |  |  |  |
|  | Rural | -5.28 (-5.82, -4.71) | 2004-2021 | -5.28 (-5.82, -4.71) |  |  |  |  |
| Colorectal | Urban | -2.82 (-3.93, -1.69) | 2004-2021 | -2.82 (-1.69, -3.93) |  |  |  |  |
|  | Rural | -1.29 (-1.94, -0.25) | 2004-2007 | -1.29 (-1.94, -0.25) |  |  |  |  |
| Liver | Urban | -5.71 (-6.56, -5.10) | 2004-2011 | -7.69 (-12.24, -5.87) | 2011-2014 | 4.85 (-2.39, 8.86) | 2014-2021 | -7.98 (-11.54, -6.30) |
|  | Rural | -3.85 (-4.56, -3.11) | 2004-2021 | -3.85 (-4.56, -3.11) |  |  |  |  |
| Pancreatic | Urban | -1.95 (-3.62, -0.23) | 2004-2021 | -1.95 (-3.62, -0.23) |  |  |  |  |
|  | Rural | 1.62 (0.17, 3.14) | 2004-2021 | 1.62 (0.17, 3.14) |  |  |  |  |
| Lung | Urban | -5.33 (-6.43, -4.16) | 2004-2021 | -5.33 (-6.43, -4.16) |  |  |  |  |
|  | Rural | -3.40 (-4.30, -2.65) | 2004-2014 | -1.70 (-2.71, 0.59) | 2014-2021 | -5.79 (-10.66, -4.11) |  |  |
| Skin | Urban | -2.46 (-4.80, 0.03) | 2004-2021 | -2.46 (-4.80, 0.03) |  |  |  |  |
|  | Rural | -2.11 (-3.65, -0.30) | 2004-2010 | -7.27 (-21.85, -2.14) | 2010-2021 | 0.82 (-1.62, 12.40) |  |  |
| Breast | Urban | -2.09 (-3.13, -1.02) | 2004-2021 | -2.09 (-3.23, -0.92) |  |  |  |  |
|  | Rural | -0.21 (-1.00, 0.60) | 2004-2021 | -0.21 (-1.00, 0.60) |  |  |  |  |
| Cervical | Urban | -0.31 (-1.99, 1.46) | 2004-2021 | -0.31 (-1.99, 1.46) |  |  |  |  |
|  | Rural | -1.26 (-2.97, 0.08) | 2004-2009 | -7.80 (-20.16, -2.26) | 2009-2016 | 7.40 (3.74, 21.29) | 2016-2021 | -6.00 (-18.42, -0.65) |
| Uterine | Urban | -8.82 (-11.49, -5.99) | 2004-2021 | -8.82 (-11.49, -5.99) |  |  |  |  |
|  | Rural | -10.36 (-14.33, -6.22) | 2004-2010 | -4.96 (-15.20, 6.25) | 2010-2021 | -13.18 (-17.03, -9.14) |  |  |
| Ovarian | Urban | -2.27 (-3.58, -0.92) | 2004-2021 | -2.27 (-3.58, -0.92) |  |  |  |  |
|  | Rural | 2.23 (1.13, 3.34) | 2004-2021 | 2.23 (1.13, 3.34) |  |  |  |  |
| Prostate | Urban | -7.22 (-9.64, -4.70) | 2004-2021 | -7.22 (-9.64, -4.70) |  |  |  |  |
|  | Rural | -2.40 (-7.77, 3.52) | 2004-2021 | -2.40 (-7.77, 3.52) |  |  |  |  |
| Bladder | Urban | -7.13 (-10.34, -4.08) | 2004-2021 | -7.13 (-10.34, -4.08) |  |  |  |  |
|  | Rural | -2.90 (-5.40, -0.34) | 2004-2021 | -2.90 (-5.40, -0.34) |  |  |  |  |
| Lymphoma | Urban | -1.05 (-2.54, 0.53) | 2004-2007 | 13.14 (1.47, 35.83) | 2007-2021 | -3.84 (-6.00, -2.65) |  |  |
|  | Rural | 1.41 (-0.22, 2.71) | 2004-2016 | 4.51 (2.98, 7.25) | 2016-2021 | -5.68 (-17.67, -0.82) |  |  |
| Leukemia | Urban | -4.75 (-5.71, -3.73) | 2004-2021 | -4.75 (-5.71, -3.73) |  |  |  |  |
|  | Rural | -3.11 (-3.62, -2.58) | 2004-2021 | -3.11 (-3.62, -2.58) |  |  |  |  |
| Others | Urban | -3.11 (-4.03, -2.14) | 2004-2021 | -3.11 (-4.03, -2.14) |  |  |  |  |
|  | Rural | -0.37 (-1.58, 0.73) | 2004-2013 | 1.87 (0.30, 7.05) | 2013-2021 | -2.84 (-9.44, -1.00) |  |  |

**Table S3. Changepoint and segment joinpoint analyses of ASMR for cancers among young people aged 15-44 years old stratified by rural and urban areas in China from 2014 to 2021**

| **Type** | **Region** | **Changepoints detected** | **Trend1 years** | **APC (%)** | **Trend2 years** | **APC(%)** | **Trend3 years** | **APC (%)** |
| --- | --- | --- | --- | --- | --- | --- | --- | --- |
| Overall | Urban | - | 2004-2021 | -4.13 (-4.90, -3.34) |  |  |  |  |
|  | Rural | - | 2004-2021 | -2.74 (-3.21, -2.26) |  |  |  |  |
| Liver | Urban | 2010, 2013 | 2004-2010 | -5.62 (-7.49, -3.70) | 2010-2013 | 2.72 (-30.67, 36.30) | 2013-2021 | -7.48 (-8.91, -6.03) |
|  | Rural | - | 2004-2021 | -3.85 (-4.56, -3.11) |  |  |  |  |
| Lung | Urban | - | 2004-2021 | -5.33 (-6.43, -4.16) |  |  |  |  |
|  | Rural | 2014 | 2004-2014 | -1.70 (-2.71, 0.59) | 2014-2021 | -5.79 (-10.66, -4.11) |  |  |
| Others | Urban | - | 2004-2021 | -5.79 (-10.66, -4.11) |  |  |  |  |
|  | Rural | 2013 | 2004-2013 | 1.87 (0.30, 7.05) | 2013-2021 | -2.84 (-9.44, -1.00) |  |  |
| Leukemia | Urban | - | 2004-2021 | -4.75 (-5.71, -3.73) |  |  |  |  |
|  | Rural | - | 2004-2021 | -3.11 (-3.62, -2.58) |  |  |  |  |
| Stomach | Urban | - | 2004-2021 | -5.38 (-6.48, -4.24) |  |  |  |  |
|  | Rural | - | 2004-2021 | -2.82 (-1.69, -3.93) |  |  |  |  |
